# Modeling solute transport in karst fissure dual porosity system and application: A case study in an arsenic contamination site

**DOI:** 10.1371/journal.pone.0234998

**Published:** 2020-06-25

**Authors:** Kuixian Huang, Xingzhang Luo, Zheng Zheng

**Affiliations:** Department of Environmental Science & Engineering, Fudan University, Shanghai, China; Sapienza Universita di Roma, ITALY

## Abstract

Fractures and fracture networks are key conduits for migration of hydrothermal fluids, water and contaminants in groundwater systems Modeling is widely used to understand the environmental risk associated with migration of pollutant for different hydrogeological conditions. In this paper, we proposed a conceptual and mathematical model of flow and transport phenomena in fractured rock systems, and applied in a arsenic contaminate site as a case study. The groundwater flow model and arsenic migration model in fissure-matrix dual system were established. The results show that the velocity of groundwater is positively correlated with inlet pressure, but not with the porosity of the fracture. When the velocity of groundwater is relatively high, arsenic is transported along with the current in a finger-like manner. The distribution of arsenic concentration on the fracture surface is heterogeneous and the phenomenon of diffusion from the fracture to the matrix is not obvious. Indeed, when the velocity of groundwater is relatively small, the arsenic moves forward slowly, the concentration distribution on the crack surface is relatively uniform, and the diffusion phenomenon from the crack to the matrix is more significant.

## Introduction

Fractured carbonate aquifers, which are subjected to different degrees of karstification, underlie a land area covering ~ 15% of the earth’s surface and supply ~ 25% of the world’s population with drinking water [1]. Arsenic pollution in groundwater is one of the hot environmental issues around the world [2, 3]. In the 1930s, native high-arsenic groundwater and its health risks were discovered in Canada [4], New Zealand [5] and Taiwan [6]. Up to now, more than 70 countries around the world have discovered native high-arsenic groundwater. About 137 million people in the world drink groundwater within arsenic concentration exceeding 10 μg•L^-1^, including 15 million in China [7]. Some researchers have established different risk assessment models to evaluate the health risks of arsenic pollution in groundwater [8, 9]. As for the migration rule of arsenic in groundwater, some studies considered that iron/manganese oxides, organic matter, and REDOX conditions are the main factors affecting the migration and transformation of arsenic in water bodies [10–13]. Local conditions/geometry strongly affect the distribution of fractures and thus porosity, permeability and elastic parameters [14, 15].

Compared with the closed and homogeneous underground aquifer system and the open surface water system, the study of pollutant migration in karst fracture groundwater system was few. In karst fracture groundwater system, karst fissure media contains lots of media forms, such as pores, cracks, karst pipes, small karst caves, etc. In addition, the pollutant may be trapped and enriched in surface karst zone where karst development is not strong and connectivity of fissure and fissure holes is not good, except in karst pipes and other media [16–18]. Therefore, it is essential to establish migration model to simulate pollutant migration in groundwater in karst fissure-matrix dual system.

Contaminants that are highly soluble in groundwater are rapidly transported via fractures in mechanically resistant sedimentary rock aquifers [19, 20]. The rapid underground horizontal flow motion existing in the fracture-like pipeline medium can be transformed into nonlinear turbulence under certain conditions, which cannot be described simply by Darcy’s law. However, it is inaccurate to generalize a complex karst aquifer into a single water-bearing pore medium and to use traditional numerical codes (e.g., MT3DMS) to carry out groundwater flow field and solute transport [21, 22]. Therefore, it is essential to propose a new method based on flow characteristic of karst fissure-matrix dual system to carry out groundwater flow field and solute transport.

In this paper, the hydrodynamic equation is used to consider the hydraulic connection between the porous medium and the pipeline medium. The exchange coefficient between the mediums is substituted into the flow equation. The dual medium model of the karst fissure-matrix system is then established. The results of water flow and solute transport will provide simulation information of pollution distribution and migration trend, which is useful to formulate accurate measures for remediation of contaminated sites.

## Study area

The present study was conducted in Zhele site (107.44° E, 25.229°N), located in Hechi city, South China, where serious arsenic contamination events have taken place in recent years. The Zhele site belongs to an abandoned arsenic chemical plant, founded in 1995 with an outdated method, operated until 2003. The pollutants remaining in the abandoned site can still pose hidden danger to the surrounding environments. The study area is located in a warm humid subtropical monsoon mountain climate with an average annual temperature of 17.8 °C. The average annual precipitation is 1377.1 mm, and the highest precipitation occurs during May–September. The topography of the region is Middle Mountain with a big slope. No specific permissions were required for these locations. The field studies did not involve endangered or protected species.

## Methods

### Mathematical equation

#### Groundwater flow

In this paper, the multi-physical field simulation software COMSOL Multiphysics (hereinafter referred to as COMSOL) is used to simulate the water flow and solute transport of the fissured matrix system [23]. The interface of "fracture flow" is adopted for water flow simulation. The flow formula of the interface of "fracture flow" is a modified form of Darcy’s law, which uses the tangential derivative to define the flow of fluid in porous media along the internal boundary representing the fracture, and is generally used to describe the flow of fluid along the internal fracture in a porous (solid) media.

Darcy’s law describes the velocity field jointly controlled by the properties of porous media, fluid viscosity, and pressure gradient, and its mathematical expression is shown in Eq (1).

$$u=-\frac{\kappa }{\mu }\nabla p$$ (1)

where *κ* is the Permeability of porous media, the unit is m^2^. *μ* is the dynamic viscosity of a fluid, the unit is kg/(m·s); p is pressure, the unit is Pa; **u** is Darcy flow velocity, the unit is m/s.

Darcy’s law is combined with the continuity equation as shown in Eq (2).

$$\frac{\partial }{\partial t}(\varepsilon \rho )+\nabla ∙(\rho u)=Q_{m}$$ (2)

where ρ is fluid density, the unit is kg/m^3^; ε- porosity of porous media; Q_m_ is mass sources, unit is kg/(m^3^·s). Symbols and units of other variables are the same as above.

The mathematical expression of the volume flow over the length of the fracture unit is shown in Eq (3) (ignoring the effect of gravity).

$$q_{f}=-\frac{\kappa_{f}}{\mu }d_{f}(\nabla_{T}p)$$ (3)

where q_f_ is volumetric flow of fluid per unit length of the fracture, the unit is m^2^/s; *κ*_*f*_ is permeability of fracture, unit is m_2_; *d*_*f*_ is pore of fracture, unit is m; ∇_*T*_ is gradient operator in tangent plane of fracture. The symbols and units of other variables are the same as above.

From the volume flow rate, q_f_ per unit length in the fracture of formula (3), the flow velocity u in the fracture is obtained as shown in formula (4).

$$u=\frac{q_{f}}{d_{f}}=-\frac{\kappa_{f}}{\mu }(\nabla_{T}p)$$ (4)

Combining material properties, Eq (3), and the continuity equation across the fracture cross-section, the pressure equation is obtained as shown in Eq (5).

$$d_{f}\frac{\partial }{\partial t}(\varepsilon_{f}\rho )+\nabla_{T}∙(d_{f}\rho u)=d_{f}Q_{m}$$ (5)

where *ε*_*f*_ is porosity of fracture. The symbols and units of other variables are the same as above. Eqs (4) and (5) together form the control equation of fissure flow.

### Solute transport

In fissure-matrix system, solute transport can be described as two processes: (1) solute transport in fissures; (2) solute diffusion from crack to matrix. The first process involves convection and diffusion, and in the second, the solute transport is mainly molecular diffusion because the permeability of the matrix is very low. The migration along the fracture is much faster than in the matrix. When the solute is transported in the crack, the diffusion from the crack to the matrix cannot be ignored, which conforms to Fick diffusion law. The mathematical expression of the diffusion from the crack to the matrix is as follows:

$$\frac{\partial \theta C}{\partial t}-\nabla ∙\theta D(\nabla C)=0$$ (6)

Mathematical equation expression of solute transport in crack:

$$\frac{\partial \theta C}{\partial t}+u∙\nabla C-\nabla ∙\theta D(\nabla C)=0$$ (7)

Where C is concentration of solutes in the study domain; t is time; θ is matrix porosity; u is flow velocity; D is molecular diffusion coefficient.

The above two forms jointly control the solute migration process in the fracture-matrix system.

### Conceptual model

#### Fissure matrix system

The model generalized as a fissure-matrix system model, which focuses on the study of fluid flow and pollutant transport. The whole area is divided into two parts. One part is the fracture network area with low water storage capacity but high permeability, which is the main transport channel of groundwater and pollutants. The other part is the matrix region, with low permeability and almost impermeable to water. The permeability of the matrix block of fractured rock mass is much lower than that of the fracture network. The fan-matrix system model simulates each fracture in the rock mass fracture network system and tries to obtain the real seepage state of each point in the fracture system. It obviously has the advantages of good plausibility and high precision.

#### Model conceptualization of fracture matrix system

To simulate the relatively real rough fracture surface, interpolation data were used in this study, which represented that the aperture a(x, y) of the fracture varied with its position, which contained the sample aperture data in the form of 100*100 matrix [24]. The synthesized data set corresponds to the aperture with a fractal dimension of 2.6. An interpolation function data is defined in COMSOL, and the aperture data is imported into the interface of the COMSOL physical field. Through linear interpolation, a rough fracture surface can be generated. To simulate a cross-fracture network, the generated fracture surface is transformed in COMSOL to obtain a set of orthogonal fractures. The fissure—matrix model is 80mm long, 50mm wide and 50mm high, as shown in S1 Fig for details. In S1(b) Fig, the blue shows the intersecting fracture surface and the remainder shows the rock matrix.

In order to study the influence of cross fractures on groundwater flow and solute transport, the velocity of groundwater in the fracture is controlled by pressure. Fracture surface 1 is set as the inlet of water flow and solute transport, and the fracture surface 2, 3 and 4 are the outlet of water flow and solute. The remaining side is no-flow boundaries. It is assumed that only the fracture surface is a water channel and the external matrix is impermeable.

#### Selection of model parameters and initial boundary conditions

The parameters used in the water flow model are generally empirical values of karst fissure dual porosity system in southwest china [24], as shown in Table 1. In the water flow model, the aperture in the horizontal and vertical directions of the fracture is calculated automatically during operation according to the interpolation function defined in COMSOL. The expression of the aperture of the horizontal fracture surface is A = data(x,y)/1000, and the expression of the aperture of the vertical fracture surface is A = data(x,z)/1000. According to the "cubic law", the permeability of the fracture is proportional to the square of the aperture of the fracture, namely $\kappa_{f1}=\frac{A^{2}}{12}$ and $\kappa_{f2}=\frac{a^{2}}{12}$.

**Table 1:** List of selected model parameters.

Parameter	Symbol(unit)	Value
fluid density	ρ(kg/m^3^)	1000
acceleration of gravity	g(m/s^2^)	9.80665
hydrodynamic viscosity	μ(Pa*s)	0.001
fracture of porosity	εf(/)	0.8

In this study, the inlet velocity was controlled by pressure, and the initial pressure of the whole model was 0. The inlet and outlet are both fixed pressure boundaries, and the inlet pressure is set as 800 Pa, 600 Pa, 400Pa, 200Pa, 100Pa, and 50Pa, respectively, and the outlet pressure is 0.

Since parameters such as diffusion coefficients are difficult to obtain in fracture media, the parameters in this study are selected from empirical values [24]. The values of solute transport module parameters are shown in Table 2.

**Table 2:** Solute transport module parameters in model.

Parameter	Symbol(unit)	Value
Diffusion coefficient of fluid in fracture	Df (m2/s)	2.00E-09
Diffusion coefficient of fluid in matrix	Dm (m2/s)	2.00E-12
Matrix porosity	θ (-)	0.25
Pollutants concentration at the inlet	C0 (mol/m3)	1

#### Grid generation

**Fig 1:**
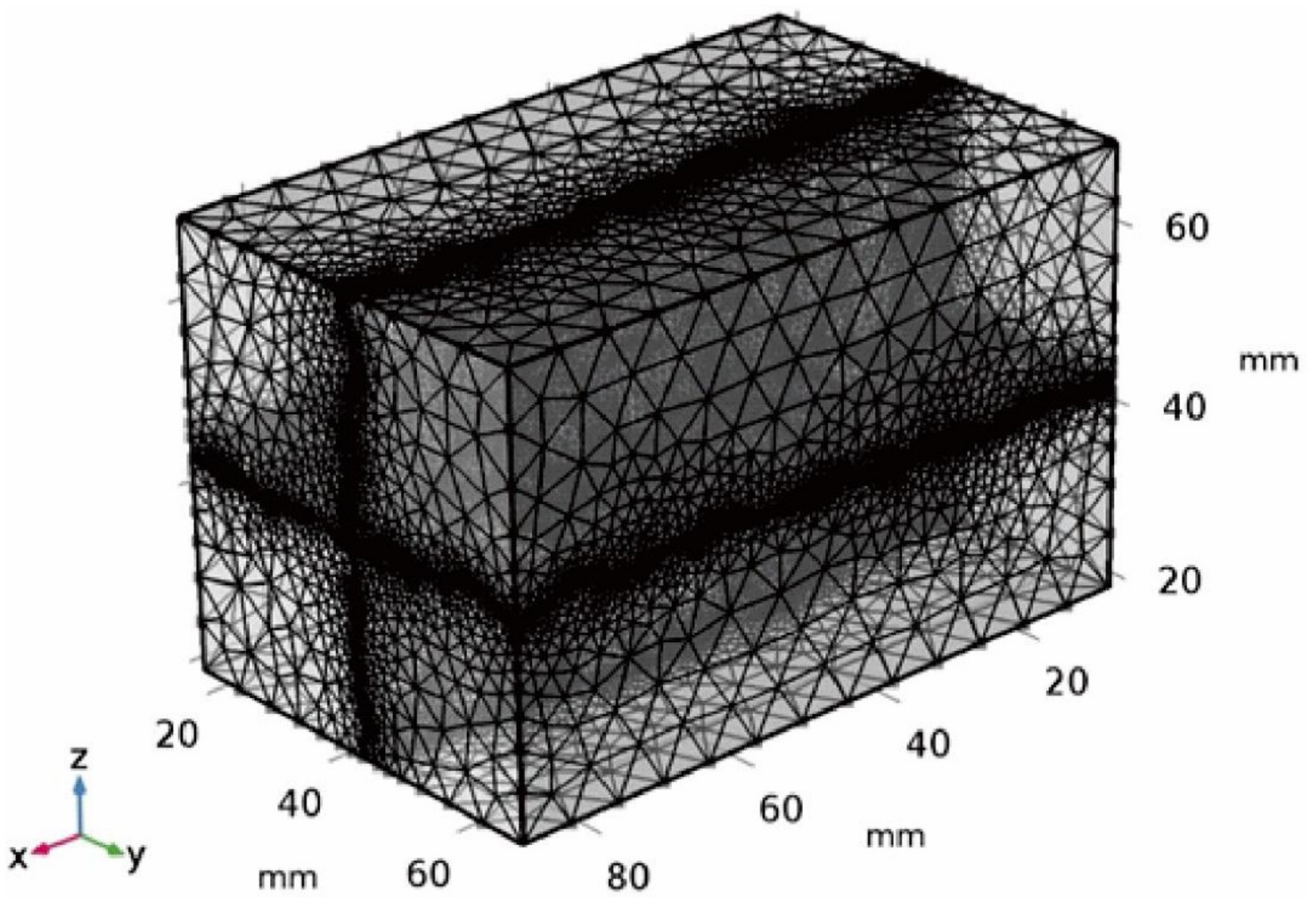
Grid generation in karst fissure dual porosity system.

In terms of grid generation of the model, the whole model domain is divided into free tetrahedrons, and the grid is encrypted at the crack surface, as shown in Fig 1. The whole model domain is divided into 325,018 tetrahedral elements, 950 boundary elements, and 18 vertex units. The smallest unit mass is 0.1996 and the average element mass is 0.6542.

## Results and discussion

### Flow model

**Fig 2:**
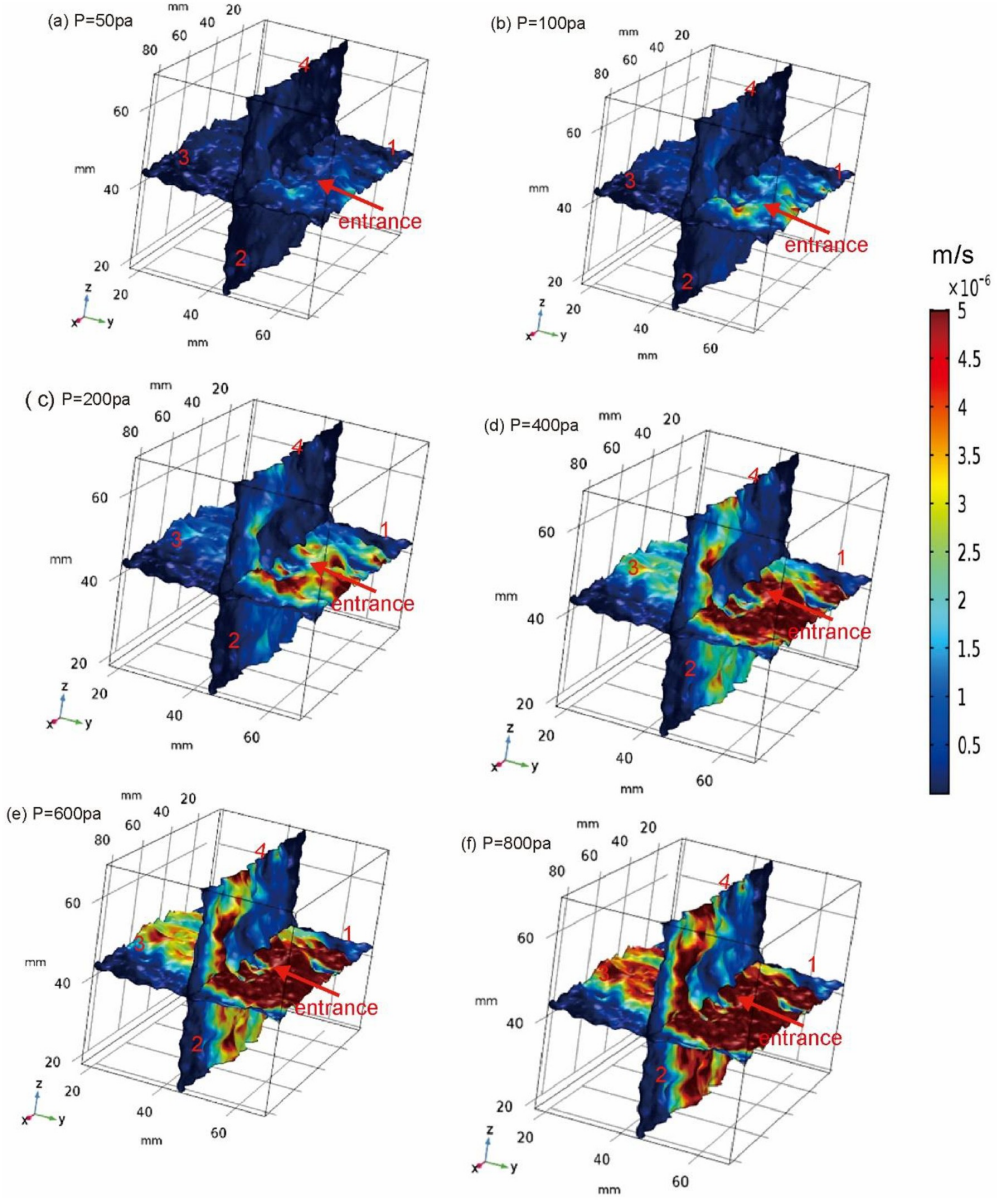
Velocity profile under different inlet pressures.

The fissure-matrix system boundary 1 is the inlet boundary, and the boundary 2, 3 and 4 are the outlet boundary. The simulated water flow results are shown in Fig 2. In Fig 2, the color represents the size of the velocity, with red having the highest velocity and the dark blue having the lowest. Apply 50Pa, 100 Pa, 200 Pa, 400 Pa, 600 Pa, 800 Pa pressure at the entrance. As the pressure increases, the velocity also increases, and the high velocity area also increases. It can be seen from the velocity distribution diagram that the water flow velocity in the horizontal fracture plane in the orthogonal fracture is slightly higher than that of the vertical fracture. The velocity distribution on the whole fracture surface is obviously heterogeneous, which is caused by the uneven velocity distribution due to the different roughness of the fracture surface.

S2 Fig shows the distribution of pressure and velocity when P = 400Pa. In S2(a) Fig, the pressure varies greatly on fracture surface 1, and the pressure difference increases where the pressure on the fracture surface fluctuates greatly. The corresponding velocity of the same part in S2(b) Fig is also large, and priority flow is generated on the fracture surface. However, in the fracture surface 2, 3 and 4, the pressure change is small, the pressure difference is smaller, and the flow rate is decreased. After calculating the average flow velocity of the entire fracture surface, the relationship between the average flow velocity and the corresponding inlet pressure is obtained, as shown in S3 Fig. It can be seen from S3 Fig that the average flow rate is linear with the pressure.

### Solute transport model

**Fig 3:**
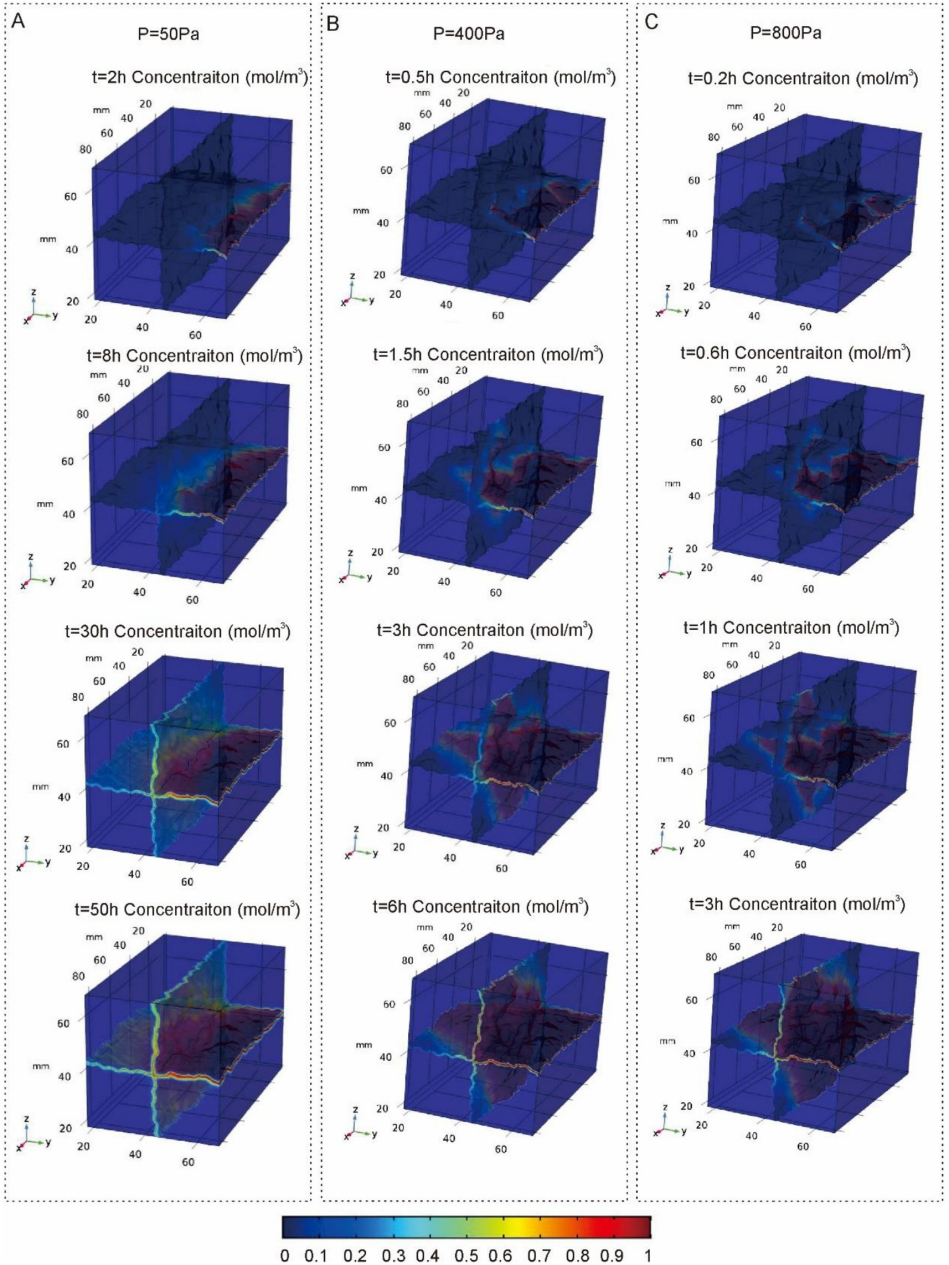
Distribution of pollutant concentration under different time at different inlet pressures (A, P = 50Pa; B, P = 400Pa; C, P = 800Pa).

Through the comparative analysis of pollutant transport processes under different inlet pressures, when P = 50Pa, the velocity of groundwater flow is small, and the pollutants move slowly towards the outlet under the action of convection and molecular diffusion (Fig 3A). During the migration process, the convection effect is not obvious and no preferential flow is formed. Under the influence of diffusion, the pollutants expand to both sides and distribute uniformly in the three outlet cracks. The reason for this phenomenon is that the diffusion in the fracture is the main force of pollutant transport at a low velocity, which exceeds the influence of the water flow changes caused by the fracture surface roughness. The diffusion is related to the flow velocity, and at a relatively low flow velocity on the fracture surface, the pollutant’s ability to invade the fracture surface is far less than its ability to transport in the direction of the flow. At the periphery of the crack, the color changes from red to blue along the direction perpendicular to the crack, indicating that the pollutant not only migrates in the crack through convection but also diffuses from the crack to the matrix through molecular diffusion.

With the increase of inlet pressure, the flow velocity in the corresponding crack increases. With the rapid migration of pollutants along with the flow, preferential flow is formed in the area with high flow velocity, while in the area with no obvious convection effect, pollutants mainly migrate slowly by molecular diffusion, and the concentration distribution of pollutants has a strong heterogeneity (Fig 3B). The concentration profile of pollutant migration also corresponds to the flow velocity profile previously simulated. When the inlet pressure is equal to 400Pa, the flow velocity is fast and the residence time of pollutants in the crack is short. Therefore, the diffusion of pollutants from the crack to the matrix is not obvious.

As a further increase in groundwater flow rate, pollutants also move rapidly (Fig 3C). The preferential flow is more pronounced and the distribution of pollutants is more uneven. The movement of the front of pollutants is closely related to the fissure shape and roughness. Generally, the front moves along the flow direction to the fracture area with large flow velocity and the main migration direction of pollutants is consistent with the dominant flow direction of the water flow.

With the increase of inlet pressure, the velocity of flow gradually increases, the high-velocity area gradually increases, and the velocity of pollutant transport is also faster and faster. In S4A, S4B and S4C Fig have not reached the outlet within 2h, while D, E, F has reached the outlet boundary within 2h. Due to the different roughness of the crack surface and the difference of the driving force, the pollutant priority flow appears in the calculation area, which reaches the outlet of the crack first in some part of the outlet.

**Fig 4:**
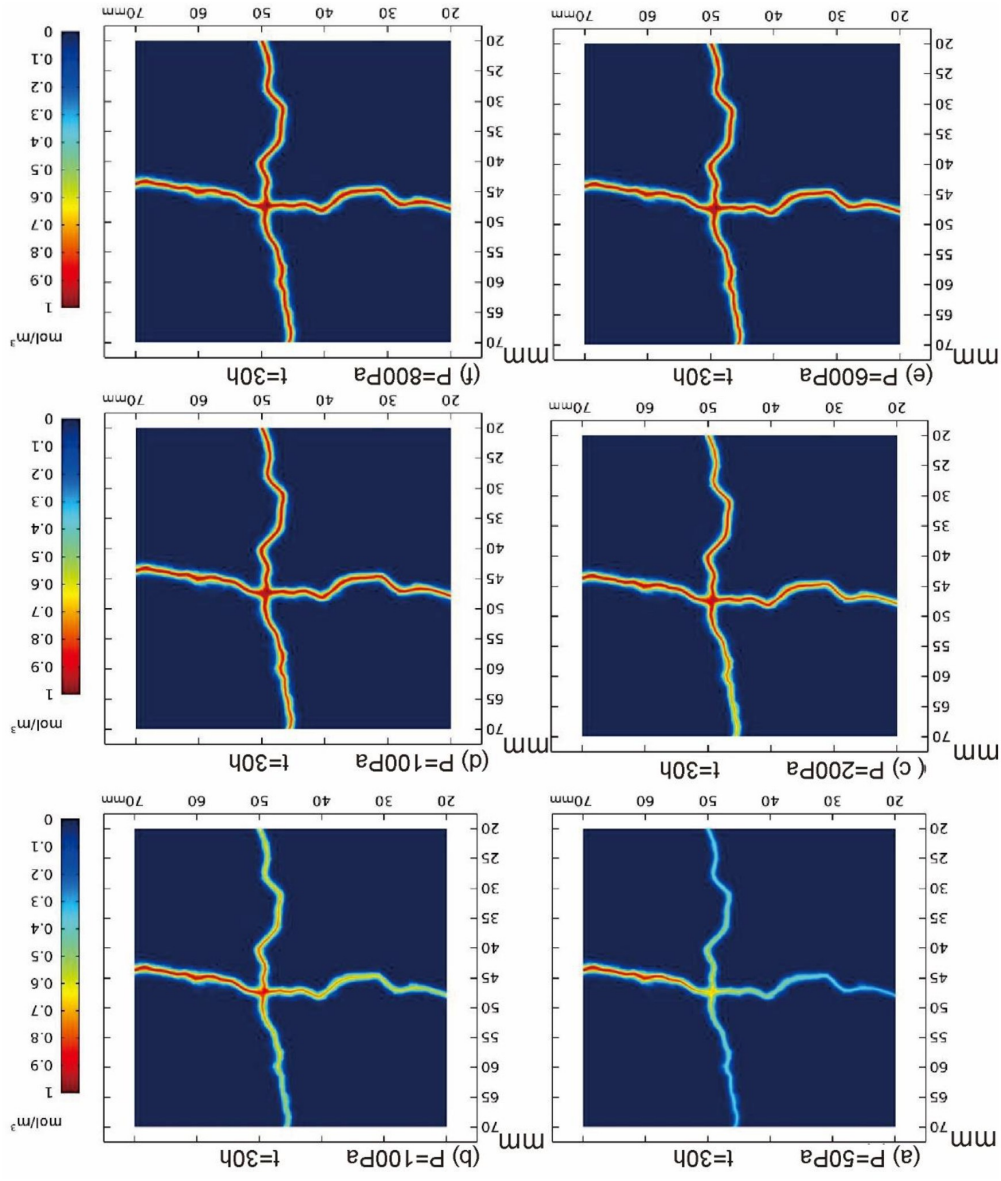
Concentration profile of X = 40mm cross section at different pressures.

Cross section of YZ at X = 40mm in the fissured matrix system is intercepted, and the position of cross section is shown in S5 Fig. Fig 4 captures the concentration distribution of X = 40mm cross section under different pressures. As shown in the figure, with the increase of inlet pressure, the transport speed of pollutants is accelerated.

In order to facilitate the comparison and quantification of the results, the concentration and time are dimensionless:

$${\text{C}}^{’}=\text{C}/{\text{C}}_{0}$$ (8)

$${\text{t}}^{’}=\text{t}/{\text{t}}_{0}$$ (9)

**Fig 5:**
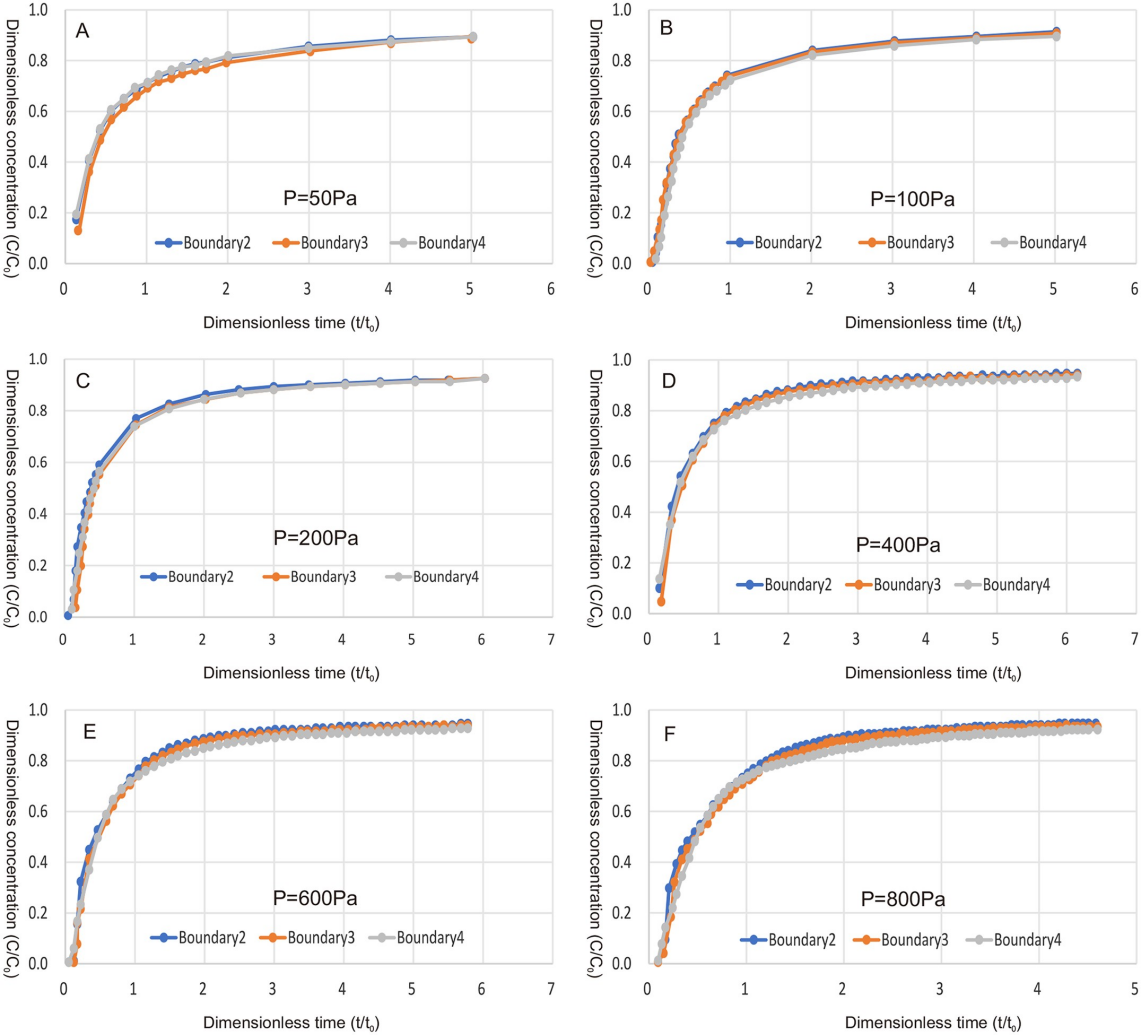
Penetration curves of three exit boundaries at different pressures.

Where t_0_ is the average retention time of water flowing through the fracture, t_0_ = L/u, L is the fracture length, and u is the average flow velocity of the fracture in the main direction of each branch. Fig 5 shows the penetration curve of each outlet crack under different pressures. It can be seen from Fig 5, under all pressure conditions, the outlet concentration of the three fracture surfaces reaches a steady state roughly when t = 5t_0_, and the concentrations of the three fracture surfaces remain basically the same. In the steady state, the concentration is 0.9mol/m^3^. When t = 2t_0_, the concentration at the outlet increases rapidly, and then the increase rate of pollutant concentration slows down significantly, indicating that the pollutant movement in the crack is delayed by the influence of matrix diffusion.

### Sensitivity analysis

The selection of model parameters will directly affect the accuracy and accuracy of the model establishment. In the parameter selection of the water flow model, only the parameter value of the porosity in the fracture is selected according to the empirical value, so its influence on the operation of the model needs to be tested. In the water flow model, the empirical value is only the porosity in the fracture. The porosity values in the fracture are selected as 0.4, 0.6, 0.8 and 1, respectively, to explore the influence of porosity in the fracture on the water flow model. Set the inlet pressure to 400Pa. The simulation results show that the change of fracture porosity has no effect on the velocity distribution, and the average flow velocity of the whole fracture model is the same under the condition that the fracture porosity values are different. The porosity of the fracture does not contribute to the velocity.

Since the selection of the solute transport model parameters in this paper is based on the empirical values, the sensitivity of the parameters should be evaluated.

### Influence of crack diffusion coefficient

In order to explore the influence of crack diffusion coefficient on pollutant transport, a model with an inlet pressure of 400Pa is selected for simulation. The crack diffusion coefficient is set at 2e-7 m^2^/s, 2e-8 m^2^/s and 2e-9 m^2^/s, respectively. The matrix diffusion coefficient is set at 2e-10 m^2^/s, and the values of other parameters remain unchanged. The ratio between crack diffusion coefficient and matrix diffusion coefficient is defined as δ, and δ is equal to 1,000, 100, 10.

$$\delta =D_{f}/D_{m}$$ (10)

**Fig 6:**
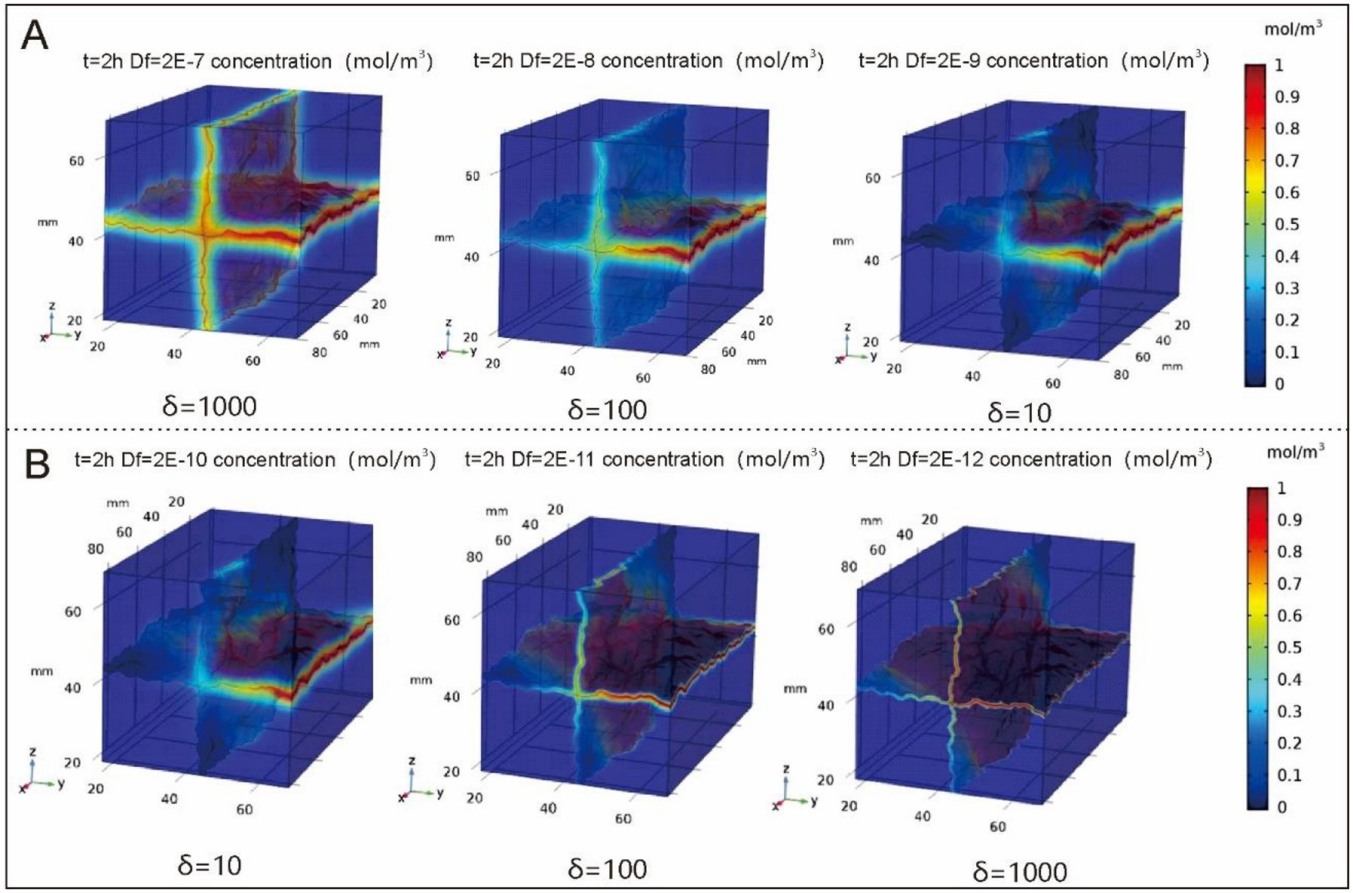
When t = 8h, the pollutant distribution diagram when delta is different.

The simulation results are shown in Fig 6. As can be seen from the figure, with the increase of δ, the transport of pollutants in the crack is faster, the influence of crack surface roughness is smaller, and the diffusion range of pollutants is larger. In the area of fracture with low velocity, the diffusion of fracture dominates, and the distribution of pollutants becomes more uniform when the diffusion coefficient of fracture is larger. When the fracture diffusion coefficient is large, the fracture diffusion coefficient has a great influence on pollutant transport, while when the fracture diffusion coefficient is small, the roughness of fracture surface and convection has a great influence on pollutant transport.

**Fig 7:**
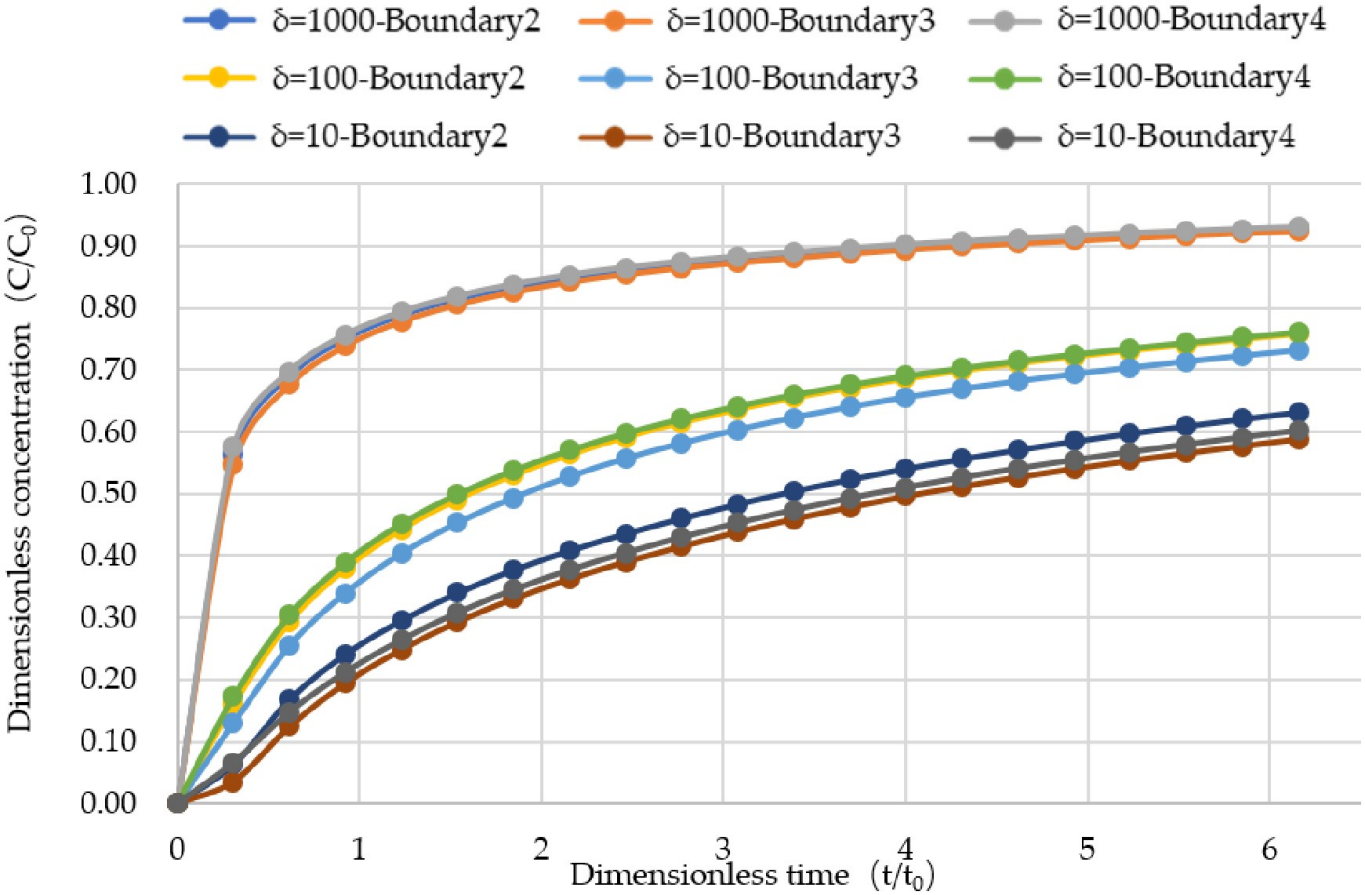
The penetrating curve of three outlets when delta is different.

Fig 7 shows the penetration curves at the outlet of the three fractures when δ takes different values. It can be seen from the figure that the larger the δ is, the earlier the penetration curve at the outlet of the fracture reaches a steady state. When δ = 1000, the penetration curve reaches a steady state at about t = 6t_0_, while the other two cases obviously take much longer, and the smaller the δ is, the longer the penetration curve will be stabilized. When δ changes from small to large, the penetration curves of the three exit boundaries gradually become uniform. When t is less than 1.5t_0_, the increase rate of pollutant concentration at the outlet is larger when δ is larger, and the convection effect is obvious. When δ is smaller, the concentration of pollutant at the outlet increases slowly, which is affected by the diffusion.

### Influence of matrix diffusion coefficient

In order to explore the influence of matrix diffusion coefficient on pollutant transport, the matrix diffusion coefficient is set at 2e-10m^2^/s, 2e-11m^2^ /s and 2e-12m^2^/s respectively. The values of other parameters remain unchanged, that is, δ is set at 10, 100 and 1000. The simulation results are shown in Fig 6B. As can be seen from Fig 6B, the matrix diffusion coefficient also has a great influence on the migration of pollutants in the fissure-matrix system. With the decrease of matrix diffusion coefficient and the increase of δ, the pollutants diffused into the matrix gradually become less, and the pollutants in the cracks become more and the contaminated area becomes larger.

**Fig 8:**
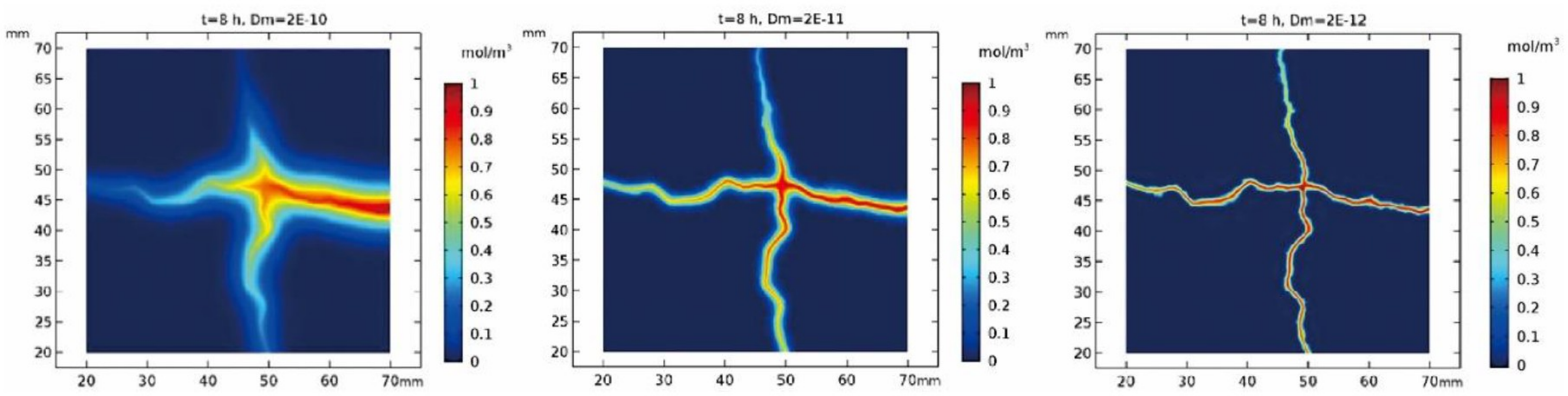
When t = 8h, take the cross section concentration diagram of delta with different values.

The position of the intercept cross section is shown in S5 Fig. When the value of D_m_ is 2e-10 m^2^/s, 2e-11 m^2^/s and 2e-12 m^2^/s, the cross-sectional concentration diagram is shown in Fig 8. In the figure, we can intuitively see that when δ = 10, the pollutant has a strong diffusion effect towards the matrix, which is caused by the small difference between the crack diffusion coefficient and the matrix diffusion coefficient. When δ is 100 and 1000, the diffusion coefficient in the crack is much larger than that in the matrix, so the diffusion of pollutants from the crack to the matrix is weak in these two cases.

**Fig 9:**
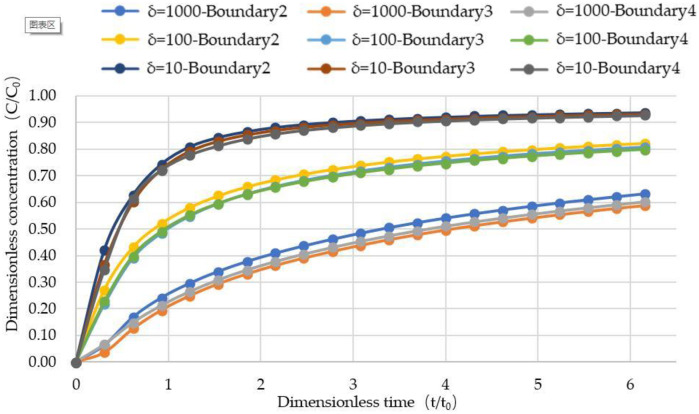
The penetration curve of three outlets under different delta values.

Fig 9 shows the penetration curve at three outlets when δ is different. It can be seen from the figure that the smaller the matrix diffusion coefficient is, the larger the δ is, and the earlier the penetration curve at the exit of the fracture reaches the steady state. when δ = 1000, the breakthrough curve reaches a steady state at t = 5t_0_, while the other two cases take longer obviously, and the diffusion coefficient matrix, the smaller the δ, the longer the time to reach stability, which is consistent with the results in the previous section. This also fully shows that the smaller the matrix diffusion coefficient, the faster the pollutant transport in the crack. Similarly, when t is less than 1.5t_0_, when δ is large, the increase rate of pollutant concentration at the outlet is large, so the convection effect is obvious; while when δ is small, the concentration of pollutant at the outlet is slowly increasing, which is greatly affected by diffusion.

The result of comprehensive sensitivity analysis shows that the solute transport result is very sensitive to both the crack diffusion coefficient and the matrix diffusion coefficient. Therefore, the correct result can be obtained only by selecting appropriate parameters when solving the model.

## Conclusion

In this study, the characteristics of arsenic pollution and arsenic migration in groundwater in the karst fan-matrix dual system in karst landform area are studied, and the groundwater flow model and arsenic migration model in the fracture-matrix dual system are established. The changes in water flow velocity and solute migration rules under different inlet pressures are discussed. The main conclusions are as follows:

1. In the study of fissure-matrix system, groundwater flow velocity is larger, the convection dominates at this time, because of the influence of the fracture surface roughness, the groundwater flow presents the strong heterogeneity and preferential flow behavior, the pollutants in finger with the current migration, the crack on the surface of the solute concentration distribution has obvious heterogeneity. When the velocity of groundwater flow is relatively small, the convection effect is not obvious, and the diffusion effect is dominant, the pollutant moves forward slowly on the whole, and the concentration distribution on the crack surface is uniform. At the same time, due to the slow transport of pollutants, the molecular diffusion from the cracks to matrix is obvious.
2. The sensitivity analysis of the water flow model and the arsenic migration model in the complex fracture-matrix dual system is carried out. The results show that the fracture diffusion coefficient and the matrix diffusion coefficient have an important influence on the migration of pollutants. The larger the diffusion coefficient, the less obvious the preferential flow of pollutant transport, until the diffusion exceeds the convection effect, and the distribution of pollutants in the fracture is more uniform. The diffusion coefficient of the matrix has a large influence on the concentration of contaminants in the matrix. The greater the matrix diffusion coefficient, the greater the concentration flux from the fracture to the matrix and the greater the concentration of contaminants in the matrix. The larger the ratio of the crack diffusion coefficient to the matrix diffusion coefficient, the easier the breakthrough curve at the exit reaches the steady state.

The results of this study show that modeling solute transport in karst fissure dual porosity system is a useful approach for provide simulation information of pollution distribution and migration trend and can aid the selection of optimal activities for contaminated site remediation. Further investigations with model that take into consideration more detailed data local geological conditions may be necessary.

## Supporting information

S1 Fig Conceptual model of fracture matrix system. (DOCX)

S2 Fig When P = 400Pa, the pressure and velocity diagram in the crack. (DOCX)

S3 Fig Average flow-pressure diagram. (DOCX)

S4 Fig Distribution of pollutant concentration at t = 2h at different inlet pressures (A, P = 50Pa; B, P = 100Pa; C, P = 200Pa; D, P = 400Pa; E, P = 600Pa; F, P = 800Pa). (DOCX)

S5 Fig Location of cross section. (DOCX)
