# Supplementary figures and images for: Modeling solute transport in karst fissure dual porosity system and application: A case study in an arsenic contamination site
Source: PLoS One. 2020 Jun 25;15(6):e0234998. doi: 10.1371/journal.pone.0234998 (PMC7316282; doi:10.1371/journal.pone.0234998)

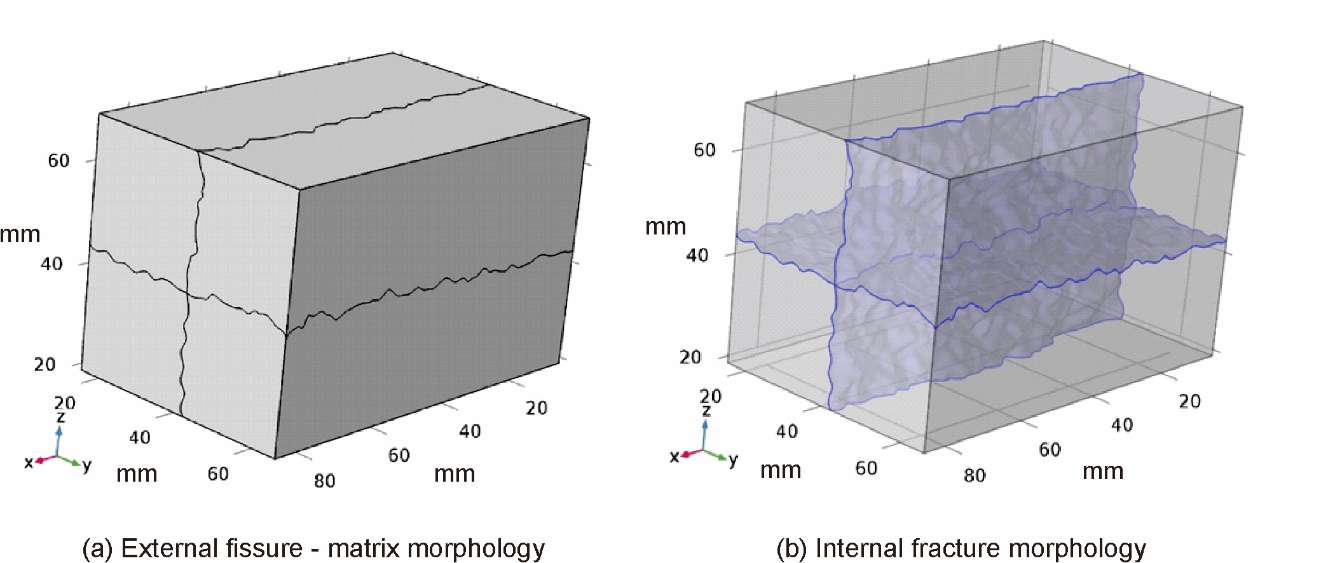

Supplement: S1 Fig — (DOCX) [file pone.0234998.s001.docx]

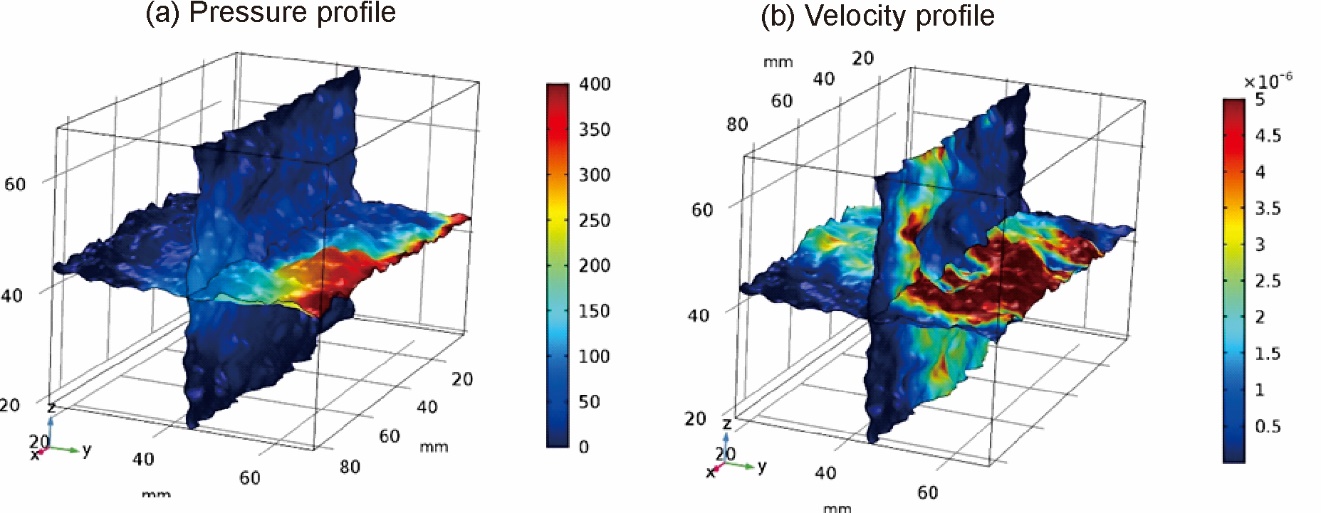

Supplement: S2 Fig — (DOCX) [file pone.0234998.s002.docx]

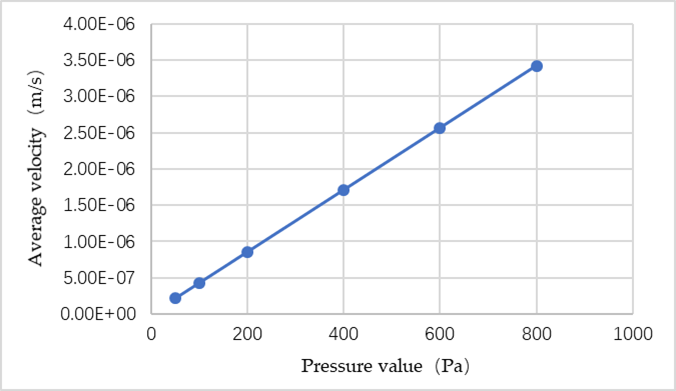

Supplement: S3 Fig — (DOCX) [file pone.0234998.s003.docx]

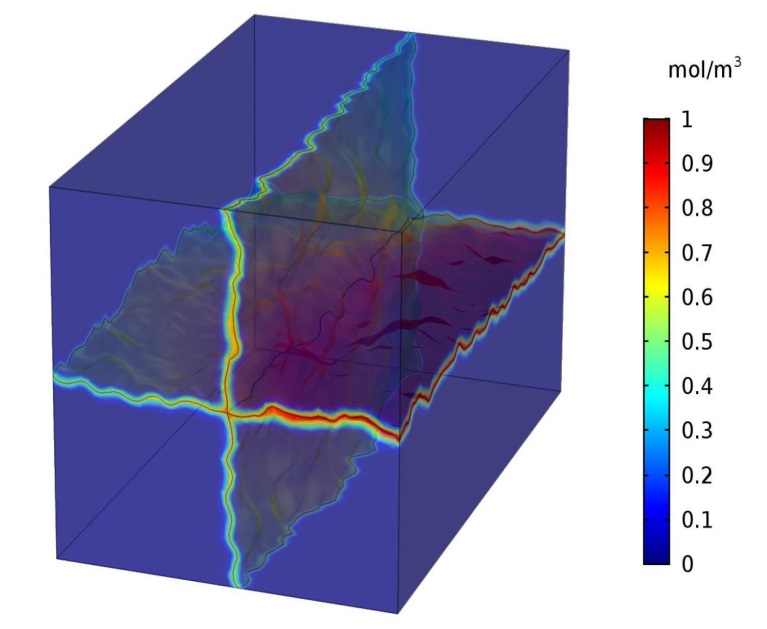

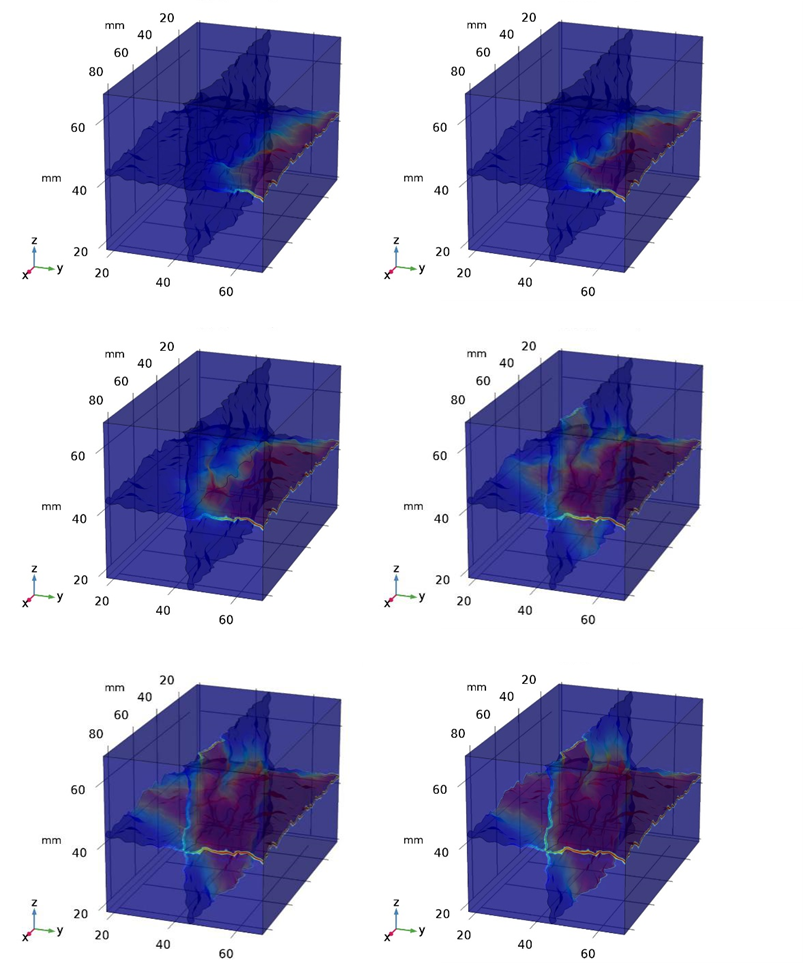


F

E

D

C

B

A

Supplement: S4 Fig — (DOCX) [file pone.0234998.s004.docx]

x

z

y

selected cross section


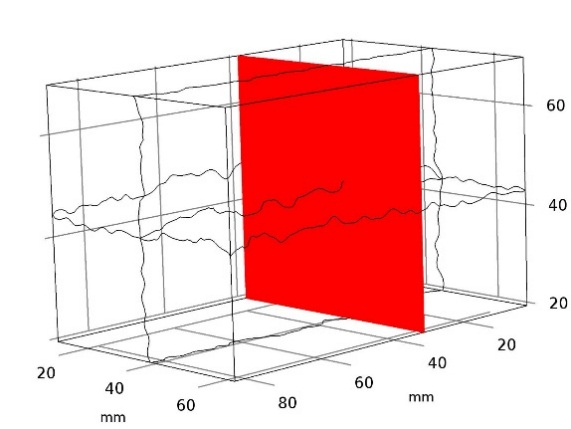

Supplement: S5 Fig — (DOCX) [file pone.0234998.s005.docx]
